# A new plasmid vector for DNA delivery using lactococci

**DOI:** 10.1186/1479-0556-7-4

**Published:** 2009-02-10

**Authors:** Valeria Guimarães, Sylvia Innocentin, Jean-Marc Chatel, François Lefèvre, Philippe Langella, Vasco Azevedo, Anderson Miyoshi

**Affiliations:** 1 Instituto de Ciências Biológicas, Universidade Federal de Minas Gerais (ICB-UFMG), Belo Horizonte – MG, Brasil; 2 INRA, UR910, Unité d'Ecologie et Physiologie du Système Digestif, Domaine de Vilvert, 78352 Jouy-en-Josas, France; 3 INRA, UR496, Unité d'Immuno-Allergie Alimentaire, Domaine de Vilvert, 78352 Jouy-en-Josas, France; 4 INRA, UR892, Unité de Virologie et Immunologie Moléculaires, Domaine de Vilvert, 78352 Jouy-en-Josas, France

## Abstract

**Background:**

The use of food-grade lactococci as bacterial carriers to DNA delivery into epithelial cells is a new strategy to develop live oral DNA vaccine. Our goal was to develop a new plasmid, named pValac, for antigen delivery for use in lactococci. The pValac plasmid was constructed by the fusion of: i) a eukaryotic region, allowing the cloning of an antigen of interest under the control of the pCMV eukaryotic promoter to be expressed by a host cell and ii) a prokaryotic region allowing replication and selection of bacteria. In order to evaluate pValac functionality, the *gfp *ORF was cloned into pValac (pValac:*gfp*) and was analysed by transfection in PK15 cells. The applicability of pValac was demonstrated by invasiveness assays of *Lactococcus lactis inlA+ *strains harbouring pValac:*gfp *into Caco-2 cells.

**Results:**

After transfection with pValac:*gfp*, we observed GFP expression in PK15 cells. *L. lactis inlA+ *were able to invade Caco-2 cells and delivered a functional expression cassette (pCMV:*gfp*) into epithelial cells.

**Conclusion:**

We showed the potential of an invasive *L. lactis *harbouring pValac to DNA delivery and subsequent triggering DNA expression by epithelial cells. Further work will be to examine whether these strains are able to deliver DNA in intestinal cells *in vivo*.

## Background

Numerous infectious agents invade the host through the mucosa to cause disease. The use of bacterial carriers to deliver DNA vaccine by oral route constitutes a promising vaccination strategy [1-3]. Most of the bacteria used to deliver DNA vaccine into mammalian cells are invasive pathogens such as *Shigella flexneri*, *Yersinia enterocolitica, Listeria monocytogenesis, Salmonella thiphymurium *or *Mycobaterium *[3-7]. Such bacteria are able to invade professional or non-professional phagocytes and deliver eukaryotic expression vectors resulting in cellular expression of the gene of interest [1,4,8]. Despite the use of attenuated strains, the risk associated with potential reversion to the wild-type (virulent) phenotype is a major concern [9].

The use of food-grade lactic acid bacteria (LAB) as DNA delivery vehicles represents an attractive alternative to the use of such attenuated pathogens and other mucosal delivery systems such as liposomes or microparticles [10]. LAB is a diverse group of bacteria transforming sugars into lactic acid. These non-pathogenic and non-invasive Gram-positive bacteria occupy different ecological niches, ranging from plant surfaces to the digestive tract (DT) of man and animals [11].

Antigen and cytokine delivery at the mucosal level by food-grade *Lactococcus lactis*, the model LAB, has been intensively investigated [12-16] (for review see [10]). In contrast to bacteria-mediated delivery of protein antigens, bacteria-mediated delivery of DNA could lead to host expression of post-translational modified antigens and therefore to the presentation of conformational-restricted epitopes to the immune system [17].

We previously developed a strategy using recombinant invasive lactococci to deliver a plasmid containing a eukaryotic expression cassette gene into epithelial cells. We demonstrated that *L. lactis *expressing *Listeria monocytogenes *Internalin A *(inlA) *gene (*LL-inlA+*) was internalized by human epithelial cells *in vitro *and enterocytes *in vivo *after oral administration of guinea pigs [18]. We also showed that *green fluorescent protein (gfp) open reading frame *(ORF) under the control of a eukaryotic promoter carried by such *LL-inlA+ *strains could be delivered into and expressed by epithelial cells [18]. These results were obtained with a large plasmid (10 kb) which is a cointegrate between an *E. coli *and a *L. lactis *replicons. During further attempts to insert antigens in this plasmid, we verified that its structure and size made difficulty not only cloning strategies but also transformation steps in lactococci.

To improve our delivery DNA strategy, we constructed a new smaller plasmid, named pValac (*Va*ccination using *l*actic *ac*id bacteria). The pValac plasmid was constructed by the fusion of: i) a eukaryotic region, containing the CytoMegaloVirus promoter (pCMV), a multiple cloning site, and the polyadenylation signal of Bovine Growth Hormone (BGH polyA) and ii) a prokaryotic region, containing the RepA/RepC replication origin for both *E. coli *and *L. lactis *and a chloramphenicol resistance gene for bacteria selection.

## Methods

### Bacterial strains and growth conditions

The bacterial strains and plasmids used in this work are listed in Table 1[18-21]. *Escherichia coli *DH5α was grown on Luria-Bertani medium and incubated at 37°C with vigorous shaking. *L. lactis *MG1363 was grown in M17 medium containing 0.5% glucose (GM17). Bacteria were selected by addition of antibiotics as follows (concentrations in micrograms per milliliter): for *E. coli*, erythromycin (100) and chloramphenicol (10); for *L. lactis*, erythromycin (5) and chloramphenicol (10).

**Table 1:** Bacterial strains and plasmids used in this work.

**Strain/plasmid**	**Characteristics**	**Source/Reference**
*E. coli *DH5α	(F^-^φ80d*lac*ZΔM15 Δ(*lac*ZYA- argF)U169 *end*A1 *rec*A1 *hsd*R17(r_k_- m_k_+) *deo*R *thi*-1 *sup*E44 λ^- ^*gyr*A96 *rel*A1)	Invitrogen
*L. lactis *MG1363	*L. lactis *subsp. *cremoris*	[19]
*LL-inlA+*	*L. lactis *expressing *L. monocytogenes inlA *gene/Ery^a ^strain	[18]
*LL-pIL253*	*L. lactis *MG1363 harboring pIL253 plasmid/Ery^a ^strain	[18]
*LL-pIL253 pValac:gfp+*	*L. lactis *MG1363 harboring pIL253 and pValac:*gfp *plasmids/Ery^a^-Cm^b ^strain	Innocentin et al., [unpublished data]
*LL-inlA+ pValac:gfp+*	*L. lactis *expressing *L. monocytogenes inlA *gene and harbouring pValac:*gfp*/Ery^a^-Cm^b ^strain	Innocentin et al., [unpublished data]
pVAX1	Expression vector containing pCMV, MCS and BGH polyA/Amp^c^-Km^d^	Invitrogen
TOPO	Cloning vector/Amp^c^	Invitrogen
TOPO:VAX1	TOPO vector containing the pCMV, MCS and BGH polyA fragment of pVAX/Amp^c^	This study
pXylT:CYT	Expression vector containing RepA/RepC replication origin/Cm^b^	[20]
pValac	Expression vector containing pCMV, MCS, BGH polyA, and RepA/RepC replication origin/Cm^b^	This study
pEGFP-N1	Expression vector containing the *gfp *gene/Amp^c^-Km^d^	BD Bioscience, Clontech
pValac:*gfp*	pValac containing *gfp *ORF inserted in the *Xba*I/*BamH*I sites/Cm^b^	This study
pIL253	High-copy number lactococcal vector/Ery^a^	[21]

^a^Ery: erythromycin resistance, ^b^Cm: chloramphenicol resistance, ^c^Amp: ampicilin resistance, ^d^Km: kanamicin resistance.

### DNA manipulations

DNA manipulations were performed as described [22] with the following modifications: for plasmid DNA extraction from *L. lactis*, TES (25% sucrose, 1 mM EDTA, 50 mM Tris-HCl pH 8) containing lysozyme (10 mg/ml) was added for 10 min at 37°C to prepare protoplasts. Enzymes were used as recommended by suppliers. Electroporation of *L. lactis *was performed as described [23]. *L. lactis *transformants were plated on GM17 agar plates containing the required antibiotic and were counted after 2-day incubation at 30°C.

### pValac vector design and construction

The eukaryotic region of pValac was obtained from the pVAX1 vector (Table 1). A 860 bp DNA fragment was generated by PCR using a polymerase with proof reading activity (Platinum *pfx *high fidelity polymerase, Invitrogen, Sao Paulo, Brazil) and the oligonucleotides CMVBglFwd (5' GGAGATCTGCGTTACATAACTTACGG 3') and BGHClaRev (5' GGATCGATTAGAAGCCATAGAGCCC 3') introducing respectively a *Bgl*II and a *Cla*I (underlined) sites in the fragment. The amplified PCR product was cloned into TOPO vector (Table 1) resulting in TOPO:VAX1 and was introduced by transformation in *E. coli *DH5α (Table 1). The integrity of the insert was confirmed by sequencing [24] using DYEnamic™ ET Dye Terminator Kit in a MEGABACE 1000 apparatus (GE Healthcare, Sao Paulo, Brazil). TOPO:VAX1 was further digested with *Bgl*II and *Cla*I restriction enzymes and gel purified (S.N.A.P. gel purification kit, Invitrogen). The prokaryotic region of pValac was obtained from the pXylT:CYT (Table 1). A 2882 bp DNA fragment was obtained after *Bgl*II and *Cla*I digestion and gel purified (S.N.A.P. gel purification kit, Invitrogen). *Bgl*II/*Cla*I-digested and purified TOPO:VAX1 and pXylT:CYT fragments were ligated using T4 DNA ligase (Invitrogen) to obtain pValac vector (3742 pb) (Table 1). pValac was established by transformation in *E. coli *DH5α and then in *L. lactis *MG1363 strains (Table 1). The integrity of the pValac sequence was confirmed by sequencing as described above.

### pValac:gfp construction

The *gfp *ORF was cloned into pValac in order to evaluate its functionality. The 726 bp *gfp *ORF, obtained from pEGFP-N1 plasmid (Table 1), was digested with *Xba*I and *Bam*HI restriction enzymes. The *gfp *ORF fragment obtained was purified (S.N.A.P. gel purification kit, Invitrogen) and then inserted into the pValac MCS using the same restriction enzymes resulting in pValac:*gfp *(4468 bp). The integrity of the *gfp *ORF was confirmed by sequencing as described above.

### Transfection assays of pValac into porcine epithelial cells

The pValac:*gfp *plasmid was assayed for GFP expression by transfection into Porcine Kidney cell line (PK15 cells). Fifty to 80% confluent PK15 cells were cultured in Dulbecco modified Eagle medium, 10% fetal calf serum, 2 mM L-glutamine (BioWhittaker, Cambrex Bio Science, Verviers, Belgium), 100 U penicillin and 100 g streptomycin. PK15 cells were transfected with 1.6 μg of pValac:*gfp*, pEGFP-N1 (positive control) or pIL253 (negative control) previously complexed with Lipofectamine 2000 (Invitrogen). pIL253 was used as a negative control due to the fate that it is an empty lactococcal plasmid; being more suitable for our next step (see below). The GFP-producing cells were visualized 48 hours after transfection with an epifluorescent microscope (Nikon Eclipse TE200 equipped with a digital still camera Nikon DXM1200). Transfection assays were performed in triplicate.

### Invasiveness assays of bacteria into human epithelial cells

To demonstrate the efficacy of pValac as a delivery vector, *LL-inlA+ *strains were transformed with pValac:*gfp *(*LL-inlA+ pValac:gfp+*) (Table 1). *In vitro *invasion assays of bacteria into human cells were performed using the human colon carcinoma cell line Caco-2 as previously described [25] with some modifications [18]. Briefly, eukaryotic cells were cultured in P6 wells plates containing 1 × 10^6 ^cells per dish in RPMI supplemented with 2 mM L-glutamine and 10% fetal calf serum. *LL-inlA+ pValac:gfp+ *or *L. lactis pIL253 pValac:gfp+ *(*LL-pIL253 pValac:gfp+*) (negative control) (Table 1) (OD600 = 0.9–1.0) were added to mammalian cells so that the multiplicity of infection (MOI) was about 10^3 ^bacteria/cell. After three hours of internalization, cells were treated for two hours with gentamicin (20 mg/ml) to kill extracellular bacteria. Fluorescent cell quantification was evaluated at 24 and 48 hours after gentamicin treatment by flow cytometry on Fluorescent Activated Cell Sorter (FACS, Becton Dickinson, France). The GFP-producing cells were visualized with an epifluorescent microscope (Nikon Eclipse TE200 equipped with a digital still camera Nikon DXM1200). Internalization and FACS assays were performed in triplicate.

## Results and discussion

### Picturing pValac

In this work, which is part of an ongoing project geared to implement safer strategies for DNA deliver and expression into eukaryotic cells, we reinforce the use of *Lactococcus lactis *as DNA delivery vehicle [18,26]. To improve our delivery DNA strategy, we constructed a new expression plasmid, the pValac.

**Figure 1:**
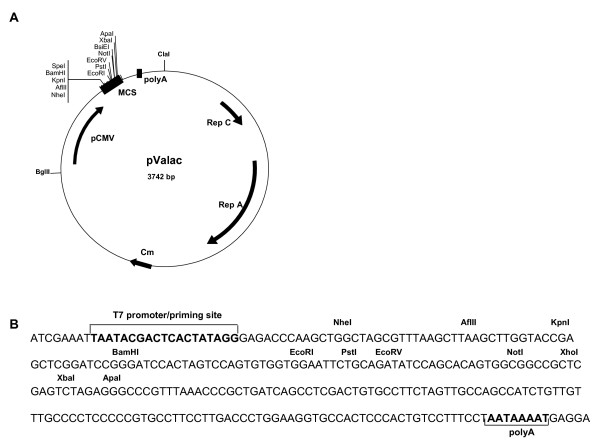
**Structure of pValac plasmid**. A: Boxes indicate: Multiple cloning site (MCS) and BGH polyadenylation region (polyA). Arrows indicate: cytomegalovirus promoter (pCMV); replication origin of *L. lactis *(Rep A) and *E. coli *(Rep C) and chloramphenicol resistance gene (Cm). *Cla*I and *Bgl*II restriction sites used to ligate eukaryotic and prokaryotic regions are showed. B: Multiple cloning site showing the T7 promoter/priming site, different restriction enzyme sites and polyA site.

pValac is depicted in Figure 1A. It harbours the eukaryotic region containing the CytoMegaloVirus promoter (pCMV), a multiple cloning site (MCS), and the polyadenylation signal of Bovine Growth Hormone (BGH polyA) needed for a gene expression by eukaryotic host cells. Its prokaryotic region contains the RepA/RepC replication origin for both *E. coli *and *L. lactis *and a chloramphenicol resistance gene (Cm) for bacteria selection. The MCS (Figure 1B), inserted between the eukaryotic promoter pCMV and the BGH polyA, carries some potential restriction enzymes that can be used to clone a gene of interest and the T7 primer binding site for its sequencing.

### Transfection assays of pValac into porcine epithelial cells

**Figure 2:**
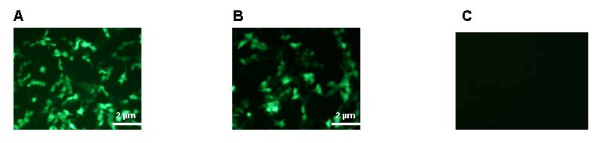
**Epifluorescent micrograph of 48 hours GFP expression by PK15 cells after transfection**. PK15 cells were transfected with pValac:*gfp*, pEGFP-N1 (positive control) and pIL253 (negative control) plasmids. A: pValac:*gfp*, B: pEGFP-N1, C: pIL253.

The pValac:*gfp *ORF was used for transfection assays into PK15 cells. Forty-eight hours after transfection with pValac:*gfp *and pEGFP-N1 (positive control), we observed comparable GFP expression in these epithelial cells (Figure 2A and 2B, respectively). No GFP expression was observed after transfection with pIL253 (Figure 2C). This result demonstrates that pValac is functional and it could be used for our further experiment.

### Invasiveness assays of bacteria into human epithelial cells

**Figure 3:**
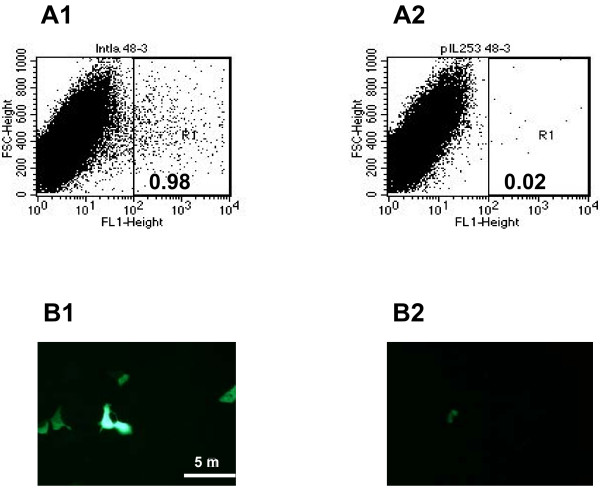
**Gene expression analysis after invasion assays**. A: *In vitro *gene transfer after 48 hours following invasion of Caco-2 cells with *L. lactis *strains carrying pValac:*gfp *assessed by FACS. A1: *LL-inlA+ pValac:gfp+*; A2: *LL*-*pIL253 pValac:gfp*+ (negative control). B: Epifluorescent micrograph of GFP expression by Caco-2 human epithelial cell line after internalization. B1: *LL-inlA+ pValac:gfp+*; B2: *LL*-*pIL253 pValac:gfp*+.

Internalization of *LL-inlA+ pValac:gfp+ *into Caco-2 cells led to GFP expression in approximately 1% of the cells 48 hours after cell invasion (Figure 3, Panels A1 and B1). A very low percentage of GFP expression was detected when the non-invasive control strain *LL-pIL253 pValac:gfp+ *was used (Figure 3, Panels A2 and B2). A MOI of 10^2 ^bacteria/cell is generally used for pathogens like *L. monocytogenes *[25] due to its virulence factors that helps bacteria to escape from vacuoles [27]. Here we used a higher MOI (10^3^bacteria/cell) for *L. lactis*, a suitable multiplicity required for an efficient internalization for these bacteria [18]. We thus demonstrate that invasive *LL-inlA+ pValac:gfp+ *were able to invade Caco-2 cells and to deliver a functional expression cassette (pCMV:*gfp*) into epithelial cells.

It is worth to note that concerning expression data, it is not surprisingly that we had comparable levels of approximately 1% using pValac:*gfp *or pVE3890 [18] since both plasmids contain the same eukaryotic genetics components, the pCMV promoter and the BGH polyA. Nevertheless, we observed that pValac is more suitable for cloning and transformation in lactococci. It is easily comprehensible that working with a small plasmid is advantageous to assemble these molecular techniques [28-31] than working with a big size plasmid.

The hypothesis for DNA delivery and expression is based on the infection of host cells by bacterial carriers: following internalization, invasive *L. lactis *is probably taken up in the vacuoles and target for degradation, thereby releasing pValac:*gfp*. Then, by an unknown mechanism, the plasmid escapes the vacuoles and reaches the nucleus where the gene (*gfp *ORF in our case) could be translated by the host cell [32-34]. Questions arise if while non-recombinant lactic acid bacteria are generally regarded as safe they still be viewed as such when invasive. *L. lactis inlA+ *survival rate was measured and showed a decrease from 4,5 log CFU/ml for 24 hours after internalisation to 2 log CFU/ml after 60 hours (data not shown). In fact, it was already suggested that *L. lactis *vaccine vectors engineered to access the cytoplasmic antigen presenting pathway are incapable of further growth in this environment [35,36]. This result suggests that, *in vitro*, these bacteria could still be regarded as safe when engineered to be invasive.

## Conclusion

Mucosal epithelium constitutes the first barrier to be overcome by pathogens during infection. The use of non-invasive bacteria for oral DNA vaccine delivery to induce intestinal mucosal immunity is a promising vaccination strategy used during the last decade. An attractive DNA vaccine strategy is based on the use of the food-grade LAB, *Lactococcus lactis*, as DNA delivery vehicle at the mucosal level.

In this sense, we constructed the pValac, a new plasmid for DNA delivery. pValac contains eukaryotic genetic elements, allowing cloning and further expression of an antigen of interest by an eukaryotic host cell as well as a prokaryotic region allowing replication and selection of bacteria. After cloning the *gfp *ORF in pValac we could show that: i) invasive *L. lactis *strains (*inlA+*) carrying pValac:*gfp *were able to enter epithelial cells and ii) after internalization, the host cells expressed the GFP protein. Therefore we could demonstrate the potential application of both plasmid and strain, to implement safer strategies for oral DNA deliver and expression into eukaryotic cells using LAB.

Further experiments have been performed to examine whether these strains are able to release enough DNA to ensure an efficient intestinal cell expression *in vivo*. In long term, an alternative strategy for DNA vaccine delivery could be achieved based on these recombinant *L. lactis *carriers.

## Competing interests

The authors declare that they have no competing interests.

## Authors' contributions

VG and SI performed the experiments of the work. VG drafted the manuscript and AM contributed to improve it. JMC and FL coordinated it. PL, VA and AM conceived the study as project leaders. All authors read and approved the final manuscript.
